# Tolerability of non-ergot oral and transdermal dopamine agonists in younger and older Parkinson’s disease patients: an European multicentre survey

**DOI:** 10.1007/s00702-020-02168-0

**Published:** 2020-05-05

**Authors:** A. Rizos, A. Sauerbier, C. Falup-Pecurariu, P. Odin, A. Antonini, P. Martinez-Martin, B. Kessel, T. Henriksen, M. Silverdale, G. Durner, K. Ray Chaudhuri, EUROPAR and the IPMDS Non-Motor PD Study Group

**Affiliations:** 1 Parkinson Foundation Centre of Excellence, King’s College Hospital London, London, UK; 2 Department of Basic and Clinical Neurosciences, Institute of Psychiatry, Psychology and Neuroscience, King’s College London, London, UK; 3 Department of Neurology, University Hospital Cologne, Cologne, Germany; 4 County Emergency Clinic Hospital, Faculty of Medicine, Transilvania University Brasov, Brasov, Romania; 5 University of Lund, Lund, Sweden; 6 University Hospital Reinkenheide, Bremerhaven, Germany; 7 Department of Neuroscience, University of Padua, Padua, Italy; 8 Center for Networked Biomedical Research in Neurodegenerative Diseases (CIBERNED), Carlos III Institute of Health, Madrid, Spain; 9 Princess Royal University Hospital Site, King’s College Hospital, Orpington, UK; 10 University Hospital of Bispebjerg, Copenhagen, Denmark; 11 Greater Manchester Neuroscience Centre, Manchester, UK

**Keywords:** Parkinson’s disease, Dopamine agonists, Tolerability

## Abstract

In older patients with Parkinson’s disease (PD), the use of dopamine agonists (DA) has been limited due to uncertainties related to their tolerability in spite of potential gains with the advent of longer acting or transdermal therapies. Comparative real-life data addressing the tolerability of DA therapy across age ranges are currently sparse. This study addressed the tolerability (Shulman criteria, continued intake of DA therapy for at least 6 months) in PD patients across several European centres treated with long-acting and transdermal DA (Rotigotine skin patch, Ropinirole extended release, or Pramipexole prolonged release) as part of routine clinical care in younger and older PD patients. A medical record-based retrospective data capture and clinical interview-based follow-up survey of patients initiating or initiated on DA treatment (short and long acting) in a real-life setting. 425 cases were included [mean age 68.3 years (range 37–90), mean duration of disease 7.5 years (range 0–37), 31.5% older age (≥ 75 years of age)]. Tolerability was above 90% irrespective of age, with no significant differences between younger and older patients. Based on our findings, we suggest that long-acting/transdermal DA are tolerated in non-demented older patients, as well as in younger patients, however, with lower daily dose in older patients.

## Introduction

Available pharmacological treatments for Parkinson’s disease (PD) include dopamine agonists (DA) which have been shown to be largely effective in numerous randomised controlled trials for both motor and some non-motor aspects (Seppi et al. 2011, 2019; Fox et al. 2018). Besides short-acting formulations, long-acting formulations have been developed and licensed which are associated with less motor complications and improved adherence to therapy (Antonini et al. 2009). In addition, we have previously reported on rates of impulse control disorder (ICD) on the same patient cohort with a focus on long-acting formulations; two oral [Ropinirole extended release (ROP-XL) and Pramipexole prolonged release (PPX-PR)] and one transdermal (TD; Rotigotine (RTG) patch) as part of the European Dopamine Agonist immediate and prolonged release Impulse Control Evaluation (DAICE) study (Rizos et al. 2016). This study was initially aimed at assessing tolerability as well as ICD rates on long-acting DA and the initial publication focussed on ICD data on advice of the reviewers at the time. ICD frequencies were significantly lower with use of RTG patch (4.9%) compared to any other assessed DA except for PPX-PR. However, data on overall clinical tolerability rate on long-acting DA or transdermal therapies in routine clinical care are sparse to date. Although many advocate not using DA in older patients, there is controversy and longer acting/TD therapies may be better tolerated (MacMahon 2003). Yet, there are no current studies addressing these issues in older patients, who are often excluded from clinical trials and not prescribed DA clinically due to tolerability concerns.

This issue is particularly relevant due to the rising incidence of PD in the older population and the numbers are predicted to further rise in future (Hirsch et al. 2016; Dorsey and Bloem 2018). Previously, one UK multicentre study reported that Cabergoline, a long-acting ergot DA, showed good tolerability in older PD patients (Appiah Kubi et al. 2003). Here, we report results on the tolerability (at least 6 months use) of non-ergot DA based on the data of the DAICE study including PR formulations (oral and TD).

## Patients and methods

### Study design

This was a data analysis of the previously published DAICE cohort, which was captured in an observational retrospective medical record- and prospective clinical interview-based survey of all PD patients in routine clinical care across different disease stages and ages who had initiated or were initiating DA treatment with a focus on PR (oral and TD) formulations (Rizos et al. 2016).

For the retropsective aspect of this report, we perused notes (where clinical consultation outcomes were usually documented) for noting the history of current DA use including side effects were available.

All patients noted to be on DA therapy were then clinically interviewed during their routine clinical follow-up consultation to assess tolerability and this was the propsective element of this report.

The collaboration included eight European centres (in the UK, Spain, Denmark and Romania) being part of EUROPAR, a European collaboration of the PD non-motor symptoms (NMS) research adopted as part of the Movement Disorder Society (MDS) Non-Motor PD Study Group.

### Ethical aspects

The retrospective medical record survey arm of this study was registered as an audit (medical record review) (Code number: AP1198-01), and the prospective component was approved as part of a longitudinal study of motor and non-motor symptoms in PD and the impact of PD treatments (the Non-motor Longitudinal International (NILS) study: UKCRN No 10084).

The study was carried out in accordance with the Declaration of Helsinki and authorised by the local ethics committees of participating centres.

### Patients

All 425 PD patients were included [PD as per the UK brain bank criteria (Lees et al. 2009)] and were on short- or long-acting DA or being initiated on DA (the choice of the DA used was at the discretion of the treating clinician). By default, therefore, patients with advanced dementia, hallucinations/psychosis, severe orthostatic hypotension were excluded as such patients would not be usually started on DA therapy). As a result, this was a convenience sample of patients willing to take part in the data survey. For our specific analysis presented in this paper, patients on longer acting/TD formulations were included. Patients on short-acting DA were also captured to account for potential changes form short to long-acting DA and to reflect a real-life cohort of patients. Patients were classified as younger (age < 75 years) or older (age ≥ 75 years), age being set as an arbitrary cut-off.

### Assessments

Demographic and disease characteristics assessed included sex, age, and duration of disease, past use of DA (dose and duration), discontinuation of past DA and reason for discontinuation, duration of current DA use, use of any other antiparkinsonian medication, and comorbid conditions. We classified tolerability according to the criteria by Shulman et al. (2000) (i.e., treatment with a DA that was maintained for a minimum of 6 months was considered “tolerated”, primary tolerability) which were subsequently adopted by Appiah-Kubi et al. (2003).

### Data analysis

Descriptive statistics (mean, standard deviation, range, proportions) were obtained for each variable as appropriate using Microsoft Excel 2010 and the Statistical Package for Social Science (version 23.0 for Mac; SPSS). To investigate if there were statistical differences in categorical variables between groups, Pearson *χ*^2^ test was applied as appropriate. A *p *value < 0.05 was considered as statistically significant (Rizos et al. 2016).

## Results

A total of 425 PD patients from 8 centres on DA treatment (initiated or already on) were included in this study [60.9% male; mean age = 68.3 years (range = 37–90); mean duration of disease = 7.5 years (range = 0–37)]. Main PD-related historical data are shown in Tabl 1. More than two-thirds (68.5%) of the patients were younger.

**Table 1:** Main demographic and Parkinson’s disease historical characteristics

Demographic characteristics	All cases (*N* = 425)
Male gender	60.9%
Mean age in years (range)	68.3 (37–90)
Mean duration of disease in years (range)	7.5 (0–37)
Median Hoehn and Yahr stage (range)	2.5 (1.0–5.0)

*N *number

Regarding different DA, 43.1% of the patients were on RTG TD patch (*n* = 183), 38.8% (*n* = 165) on ROP-XL, and 17.9% (*n* = 76) on PPX-PR. PPX-IR was taken by 105 and ROP-IR by 43 patients. 135 patients took more than 1 type of DA until the end of the observed period. In some patients, medications were changed to PR DA during the course of the observation period. 48 patients used more than 1 DA at the same time and could have been on a short- as well as a long-acting preparation (e.g. oral DA and transdermal RTG), although after a variable period treatment appeared to have standardised to a single DA.

### Primary tolerability in long-acting dopamine agonists

**Fig. 1:**
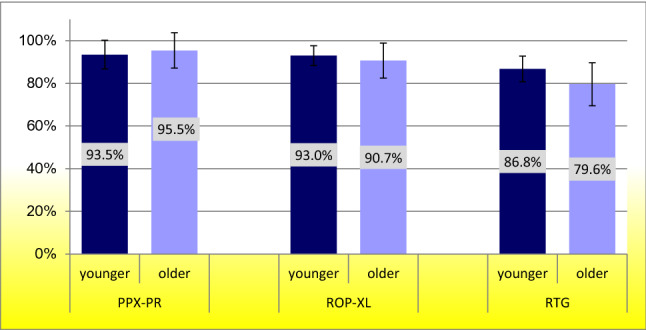
Primary tolerability rates in younger (< 75 years) and older (≥ 75 years) patients. *PPX-PR *pramipexole prolonged release, *ROP-XL *ropinirole extended release, *RTG *rotigotine; *χ*^2^ test, *p* > 0.05

Tolerability was 84.4% for patients receiving RTG (86.8% for younger PD patients, 79.6% for older PD patients), 92.3% for patients treated with ROP-XL (93.0% for younger PD patients, 90.7% for older PD patients), and 94.1% for PPX-PR (93.5% of younger PD patients, 95.5% of older PD patients). We did not find any significant differences of tolerability rates between the younger or older PD patients on any of the three different medications (Fig. 1).

Figure 1 and Table 2 show the primary tolerability rates as well as the mean dose for the respective drugs. Rates of the main causes for discontinuation regardless of the duration of prior treatment are shown in Table 3.

**Table 2:** Total (< 75 years and ≥ 75 years) tolerability rates and mean dose for prolonged release dopamine agonists

	PPX-PR	ROP-XL	RTG
Tolerability (total group)	94.1%	92.3%	84.4%
Mean dose total group	2.9 mg	12.5 mg	8.4 mg
Mean dose (< 75 years)	3.2 mg	13.7 mg	8.8 mg
Mean dose (≥ 75 years)	2.3 mg	9.2 mg	7.8 mg

*PPX-PR *pramipexole prolonged release, *ROP-XL *ropinirole extended release, *RTG *rotigotine

**Table 3:** Main side effects of DA leading to discontinuation (several possible in the same subject)

	PPX-PR (*N* = 76)		ROP-XL (*N* = 165)		RTG (*N* = 183)	
Age range	< 75 years (*N* = 52)	≥ 75 years (*N* = 24)	< 75 years (*N* = 117)	≥ 75 years (*N* = 48)	< 75 years (*N* = 122)	≥ 75 years (*N* = 61)
Skin reaction (%)	NA	NA	NA	NA	9.8	4.4
Lack of effect (%)	0	0	1.8	0	5.5	3.8
Somnolence (%)	0	1.3	1.2	0.6	0.5	0.5
Hallucinations (%)	1.3	2.6	2.4	1.8	1.1	1.1
Confusion (%)	0	1.3	1.2	0.6	0.5	1.6
ICD (%)	0	2.6	3.6	1.2	1.6	0.5

*PPX-PR *pramipexole prolonged release, *ROP-XL *ropinirole extended release, *RTG *rotigotine, *ICD *impulse control disorder, *N *number, *DA *dopamine agonists

## Discussion

In this real-life observational multicentre survey, we report on tolerability rates of DA treatment in a real-life setting.

Our results indicate that longer acting DA are well tolerated regardless of the age of the patients with an overall tolerability ranging from 84.4% in the RTG arm, 92.3% in the ROP-XL arm to 94.1% for PPX-PR, the trend being the highest for PPX-PR in older subjects (> 75 years, 95.5%). We did not find any statistically significant differences regarding tolerability between the age groups on any drug or between patients on any of those three drugs.

The reported tolerability rate, therefore, is good compared to previously reported success rates among the very elderly (above age of 80 years) of 40% for PPX and 31% for ROP (Shulman et al. 2000).

Tolerability is particularly noted for the extended release preparations even on the older population and supports the observations made by Grosset et al. (2004) in a multicentre study of adherence to oral therapy where the best concordance was reported with once a day longer acting therapies. However, the mean dose was considerably lower in older patients (approximately 30% for PPX and ROP, and 12% for RTG), therefore, DA are well tolerated in older patients but the dose range used may need to be lower based on personalised needs.

There are several limitations of this observational study. The data need to be considered in the context of a real-life observational study and as such there are obvious methodological limitations in relation to comparison of side effect issues such as ICDs particularly as the numbers of subjects in the treatment arms are different and patient population cannot be matched as this was a real-life data capture. In addition owing to the fact that the patients included are those where DA was started at clinicians’ discretion, there is an obvious bias in the exclusion of demented patients or other relative contra-indications to DA use. Nevertheless, we believe this observational report has collected important data in relation to tolerability for the first time from a multicentre study the strength of the work being collection of data from age groups of PD patients normally excluded from clinical trials (older patients (≥ 75 years). Furthermore, this was an open-label observation of real-life clinical practice generated datasets using a common data sharing network. As such the three groups of patients on PR DA and RTG TD patch could not be matched in terms of prior exposure to DA or overall levodopa equivalent daily dose.

In conclusion, the results of this international prospective observational multicentre survey of current clinical practice highlight that long-acting oral DA as well as TD DA are well tolerated in elderly PD patients without dementia although skin reactions may complicate the usefulness of TD DA. Another fundamental criticism may be “why use DA in older patients?” However, the concept of personalised medicine has been revitalised recently and the “circle of personalised medicine” incorporates several enablers and in some situations, therefore, use of DAs in older age may well be justified (Titova and Chaudhuri 2017). For example, levodopa phobia may necessity use of DA if in an older population and in a small subgroup of patients. DA may indeed be better tolerated than levodopa (Titova et al. 2018). Finally, DA such as PPX may be useful either as initiating treatment or as adjunct in older PD with severe depression or anxiety where PPX for instance may be preferable and is recommended by the MDS evidence-based committee (management of non-motor symptoms of PD) (Seppi et al. 2019; Chaudhuri et al. 2006, 2007). Healthy ageing is also common and as such lifespan of PD patients have increased and many patients also may choose to start treatment on a DA (personality aspect of the circle of personalised medicine). This work was not aimed at any recommendations for management in older patients based on this as this was not a randomised controlled study. However, such a study in real life is virtually impossible to perform logistically and as such this observational data may serve as reassurance to clinicians who may wish to prescribe oral DA in older patients as part of personalised medicine delivery.

## References

[CR1] Antonini A, Tolosa E, Mizuno Y, Yamamoto M, Poewe WH (2009). A reassessment of risks and benefits of dopamine agonists in Parkinson's disease. Lancet Neurol.

[CR2] Appiah-Kubi L, Nisbet A, Burn DJ (2003). Use and tolerability of cabergoline in young and older people with Parkinson’s disease: a multicentre observational study. J Appl Res.

[CR3] Chaudhuri KR, Martinez-Martin P, Schapira AH, Stocchi F, Sethi K, Odin P (2006). International multicenter pilot study of the first comprehensive self-completed nonmotor symptoms questionnaire for Parkinson's disease: the NMSQuest study. Mov Disord.

[CR4] Chaudhuri KR, Martinez-Martin P, Brown RG, Sethi K, Stocchi F, Odin P (2007). The metric properties of a novel non-motor symptoms scale for Parkinson's disease: results from an international pilot study. Mov Disord.

[CR5] Dorsey ER, Bloem BR (2018). The Parkinson pandemic—a call to action. JAMA Neurol.

[CR6] Fox SH, Katzenschlager R, Lim SY, Barton B, de Bie RMA, Seppi K (2018). International Parkinson and movement disorder society evidence-based medicine review: update on treatments for the motor symptoms of Parkinson's disease. Mov Disord.

[CR7] Grosset K, Needleman F, Macphee G, Grosset D (2004). Switching from ergot to nonergot dopamine agonists in Parkinson's disease: a clinical series and five-drug dose conversion table. Mov Disord.

[CR8] Hirsch L, Jette N, Frolkis A, Steeves T, Pringsheim T (2016). The incidence of Parkinson's disease: a systematic review and meta-analysis. Neuroepidemiology.

[CR9] Lees AJ, Hardy J, Revesz T (2009). Parkinson's disease. Lancet.

[CR10] MacMahon DG (2003). The initial drug treatment of older patients with Parkinson's disease—consider an agonist, but don't demonise dopa. Age Ageing.

[CR11] Rizos A, Sauerbier A, Antonini A, Weintraub D, Martinez-Martin P, Kessel B (2016). A European multicentre survey of impulse control behaviours in Parkinson's disease patients treated with short- and long-acting dopamine agonists. Eur J Neurol.

[CR12] Seppi K, Weintraub D, Coelho M, Perez-Lloret S, Fox SH, Katzenschlager R (2011). The movement disorder society evidence-based medicine review update: treatments for the non-motor symptoms of Parkinson's disease. Mov Disord.

[CR13] Seppi K, Ray Chaudhuri K, Coelho M, Fox SH, Katzenschlager R, Perez Lloret S (2019). Update on treatments for nonmotor symptoms of Parkinson's disease-an evidence-based medicine review. Mov Disord.

[CR14] Shulman LM, Minagar A, Rabinstein A, Weiner WJ (2000). The use of dopamine agonists in very elderly patients with Parkinson's disease. Mov Disord.

[CR15] Titova N, Chaudhuri KR (2017). Personalized medicine in Parkinson's disease: time to be precise. Mov Disord.

[CR16] Titova N, Levin O, Katunina E, Ray Chaudhuri K (2018). ‘Levodopa Phobia’: a review of a not uncommon and consequential phenomenon. npj Parkinson's Disease.

